# Nutrients resorption and stoichiometry characteristics of different-aged plantations of *Larix kaempferi* in the Qinling Mountains, central China

**DOI:** 10.1371/journal.pone.0189424

**Published:** 2017-12-15

**Authors:** Yajun Chang, Naiwei Li, Wei Wang, Xiaojing Liu, Fengfeng Du, Dongrui Yao

**Affiliations:** Jiangsu Key Laboratory for Bioresources of Saline Soils, Institute of Botany, Jiangsu Province and Chinese Academy of Sciences, Nanjing Botanical Garden Mem. Sun Yat-Sen, Nanjing, China; Tennessee State University, UNITED STATES

## Abstract

Elucidating the stoichiometry and resorption patterns of multiple nutrients is of essential importance to holistically understanding plant nutrition and biogeochemical cycling. Although many studies on ecological stoichiometry have been carried out, surprisingly few of them were simultaneously done on the investigation of both nutrient resorption efficiency and stoichiometry for different-aged plantations of a perennial tree. Here, both green and senesced leaf samples were collected from four *Larix kaempfer* plantations aged of 8, 15, 22, and 32 years in the Qinling Mountains to examine nutrients resorption efficiency and stoichiometry characteristics. The results suggested that the nutrient concentrations of N, P, K, Ca, Mg, Al, and Fe in both green and senesced leaves of *L*. *kaempferi* (a deciduous conifer tree) did not show a regular change trends along the plantation ages in the Qinling Mountains. The concentrations of the most nutrients examined, except for Fe, in the green leaves were relatively lower than or close to the required physiological concentrations, suggesting a relative limitation of multiple nutrients exists in *L*. *kaempferi* for its above-ground biomass growth. The rank order of resorption efficiencies of four key nutrients (N, P, K, and Mg) was K (80.89%) > N (67.42%) > P (65.34%) > Mg (41.16%), whereas the nutrient Ca and Fe tended to accumulate in senesced leaves. Overall, the nutrient resorption efficiency of all examined elements did not exhibit a regular trend corresponding to the change of the plantation ages in *L*. *kaempferi*, but it was positively related to the nutrient concentrations in green leaves. The mean C:N and C:P ratios in the green and senesced leaves were significantly higher than those reported globally (on average). By contrast, the N:P ratio, at <14, was not only much lower than that reported for both China’s flora and globally (on average), but it did suggest that the N nutrient limits growth of *L*. *kaempferi* in these plantations. Taken together, the results of this study are of substantial interest and value to forest managers and for the sustainable development of the Qinling forest ecosystems.

## Introduction

Deciduous trees play an important role in the vital process of nutrient cycling in a forest ecosystem, namely because of their strong internal and external circulation of nutrients that occurs mainly in autumn. The internal circulation, primarily in the form of nutrient resorption or retranslocation from senescing tissues, is an important strategy for a perennial plant to satisfy its annual nutrient requirements [1–3], or to overcome nutrient limitations in nutrient-poor ecosystems [1, 4–6]. Nutrient resorption affects nutrient balance, litter decomposition, plant competition etc. in ecosystem processes [5, 7–8]. Usually, nutrient resorption can be quantified in two ways, as resorption proficiency (RP) and resorption efficiency (RE) [1, 5]. The former has been defined as the terminal nutrient concentration found in the senesced leaves [5], whereas the latter RE, which represents the percentage reduction of a nutrient concentration between the green and senesced leaves, is best suited for quantifying the relative degree by which plants conserve the nutrients invested in their foliage [5].

Plants capable of high nutrient resorption have a high ability to reuse their internal nutrients, rather than losing more of them via litter [9]. Nonetheless, because nutrients contained in senesced leaves are eventually released into the forest soil, they have a crucial role in maintaining soil fertility and ecosystem productivity [5, 8, 10]. In other words, a plant characterized by a high nutrient resorption could produce relatively low-quality litter, which could affect the external circulation of nutrients by influencing the decomposition rate of litter [10, 11]. Thus, better cognition of nutrient resorption patterns is crucial for a holistic understanding of plant nutrition and biogeochemical cycling. However, many extrinsic and intrinsic variables including environmental and biological factors affect nutrient resorption capacity of plants in the field [6, 12]. To date, most studies on plant nutrient resorption have been focused on inter-plantations, but not intra-plantation variability [6–8, 13, 14]. Moreover, most researches have only addressed patterns of N and P resorption, whereas the resorption patterns of other nutrients have seldom been reported [7, 8, 13, 14].

The balance of all nutrients in plant tissues is of particular importance for plant growth [15]. Except for essential nutrient C, N and P, mineral elements mainly including K, Ca, Mg and Fe can also limit plant growth [16]. Effective resorption of the mineral elements before leaves are shed provides an important mechanism for conservation [17, 18]. On the other hand, as a key factor of causing decline of forest productivity and recession of forest, Al usually affects the normal uptake of mineral elements in plants and further causes the plants abnormal growth and development [19]. Therefore, it is an important aspect to measure Al content in leaves for precisely understanding nutrient resorption efficiency in conifers.

Ecological stoichiometry, which approaches ecological questions by asking how the balance of multiple elements as required by organisms affects their biotic and abiotic interactions, provides a new perspective for studying ecosystem processes at different scales or levels [20, 21]. According to ecological stoichiometry theory, it is essential for their healthy growth to maintain sufficient contents and stable proportions of multiple nutrients in the tissues of plants [22–24]. Although numerous ecological stoichiometric studies have been carried out, few of them apparently have examined both nutrient resorption efficiency and stoichiometry among plantations differing in age for one or more perennial plants [25, 26].

The Qinling Mountains, located in the Yellow River basin of northern China that was historically home to deciduous broadleaf forests, support a rich variety of wild flora and fauna. However, after 1970s, a large area of typical natural secondary forest was cut from the western region of the Qinling Mountains (32~35°E, 102~105°N); in its place many non-native fast-growing coniferous species, including *Larix kaempferi*, were artificially planted to increase the economic benefits from the land. *L*. *kaempferi* prefers to cool or cold climate, is suitable for cultivation in the middle mountainous area above 900 m altitude, and shows wide adaptability in China. To date, studies have been done about the effects of *L*. *kaempferi* on soil physical and chemical properties and soil nutrients. Li *et al* (2016) found that the introduction of *L*. *kaempferi* can significantly reduce soil pH of brown soil in Central China [27]. Shi *et al* (2016) noticed that the litter of *L*. *kaempferi* had a negative impact on the nutrient cycling of acid laterite soil in Dalaoling Nature Reserve [28]. In addition, *L*. *kaempferi* was reported to show an excellent growth behavior on yellow or yellow-brown soil in subtropical regions of China [29]. However, there is still no report on stoichiometry and resorption efficiency of the species, which is hindering to perceive their possible effects on the nutrient cycling and soil fertility of the local ecosystem.

The present study investigated the stoichiometry and resorption patterns of eight nutrients (C, N, P, K, Ca, Mg, Al, and Fe) in *L*. *kaempferi* plantations of four different ages in the Qinling Mountain ecosystems, China. We addressed the following questions: (1) Do the nutrient concentration and resorption efficiency show regular changes as plantation ages? (2) What elements are the relative limiting nutrients for *L*. *kaempferi* growth in the Qinling Mountains? (3) What’s the difference in resorption patterns among all the nutrients? (4) Might the presence of *L*. *kaempferi* exert effects on the nutrient cycling and soil fertility of the local ecosystem? Finally, we made practical recommendations for the forest management and the sustainable development of the Qinling Mountain ecosystems.

## Material and methods

### Site description

The study site was located in the Western Qinling Mountains (32~35°E, 102~105°N), which are 500~2500 m in elevation and are considered as the South gate of Gansu Province, China (S1 Fig). This region occupies a warm-temperate zone, and semi-humid continental monsoon climate. The maximum and minimum mean annual temperatures are 39.1°C in July and –22.4°C in January, respectively (data from 2010 to 2014), with a mean annual temperature of 12.1°C. The annual precipitation is 757 mm, and c. 70~80% of the rainfall occurs during autumn. The mean annual pan-evaporation is 1012.2 mm. The frost-free period lasts 120~218 days per year. We studied four *L*. *kaempferi* plantations that differed in plantation ages but shared a homogeneous geographical location and elevation (Table 1). The soils of all these plantations are brown-colored in type, and loam or light loam in texture, with a pH of 5.10~5.82. The detailed chemical properties of each plantation soil are shown in Table 2.

**Table 1:** Ecosystem characteristics of four *L*. *kaempferi* plantations aged 8, 15, 22, and 32 years.

Stand age (yr)	Longitude	Latitude	Elevation (m)	Aspect	Slope	Average DBH (cm)	Average Height (m)	Density (tree/ hm^2^)	Coverage
8	105°53.58′	34°08.91′	1722	SE64	42°	9.55	7.50	1733	0.85
15	105°53.97′	34°08.92′	1594	NW55	33°	10.67	10.50	1294	0.72
22	105°53.94′	34°08.66′	1582	SE35	33°	16.08	17.00	782	0.69
32	105°53.99′	34°08.05′	1586	NW15	42°	38.67	23.00	686	0.60

**Table 2:** Soil chemical properties of four *L*. *kaempferi* plantations aged of 8 15, 22, and 32 years.

Stand Age (yr)	Soil texture	Soil pH	SOC (%)	Total N (g·kg^-1^)	Total P (g·kg^-1^)	Available N (mg·kg^-1^)	Available P (mg·kg^-1^)
8	loam	5.70±0.01a	60.21±0.40d	0.76±0.00c	0.60±0.04c	59.11±6.24c	12.34±0.11c
15	light loam	5.10±0.02b	114.51±1.22a	4.21±0.01a	1.39±0.03a	159.48±6.55a	30.35±1.00a
22	light loam	5.82±0.03a	82.46±0.73c	2.44±0.03b	0.62±0.00c	108.34±4.19b	13.76±0.97c
32	loam	5.23 ±0.05b	87.34±5.00b	2.33±0.05b	0.70±0.01b	109.52±6.85b	15.98±0.25b

*Note*: Different letters indicate a significant difference at a level of α = 0.05.

### Leaf collection and chemical analysis

We selected four *L*. *kaemp*feri plantations with different ages of 8 15, 22, and 32 years in the Qinling Mountains, 3 replicates for each plantation. The basic ecosystem characteristics of each plantation were shown in Table 1. Considering the influence of sampling size to the nutrient concentration and resorption, 15 trees were selected and marked for each plantation based on the similar growth height of *L*. *kaempferi*. Mature green leaves were collected from 8–10 trees labeled previously in mid-July 2014 (the peak period of growth). We picked the leaf samples from the northern, southern, eastern and western branches of the middle part of the tree canopy and mixed together, 15 replicated leaf samples were totally collected for each plantation.

In the autumn (late September to early October) of 2014, a total of fifteen 0.5 m × 1.0 m litter traps consisting of nylon mesh (1.0 mm mesh size) were arranged under the labeled trees of each plantation, and were placed 25 cm above the ground to collect newly senesced leaves (i.e., fresh litter). We collected the fresh litters in the four *L*. *kaempferi* plantations at an interval of 5 days (5–30 days) from late September to early October, 2014. In this way, we obtained nine replicated samples each time for each plantation.

Both the green mature leaf and senesced leaf samples were immediately taken to the laboratory after collection, oven-dried at 60°C for at least 48 h, and finely ground using an electric-mill (FSJ-A05B1, Bear, China). The concentrations of nitrogen and carbon were analyzed by using the Fisons EA-1108 CHNS-O Elemental Analyzer (Fisons, Milan, Italy). For six elements of P, K, Ca, Mg, Al and Fe, 0.5 g dry samples were ashed at 550°C in a muffle furnace for 3 h and then digested overnight with 10 ml of 2.8% nitric acid. Total volume of each sample was brought to 50 ml by using ultra pure distilled water. The ensuing digested solutions were analyzed for P, K, Ca, Mg, Al and Fe by an atomic absorption spectrophotometer (TAS-986, PGENERAL, Beijing, China).

### Soil sampling and chemical analysis

Soil samples were collected at a 0–20 cm depth under the canopy of sampling trees in each plantation, and three mixed samples were taken for each plantation. These soil samples were kept in plastic bags and then transferred to the laboratory, where the samples were air-dried and then passed through a 2-mm sieve. Soil pH was measured using soil-water ratio of 1:5 (w:v) and a glass electrode pH-meter (Model 9107 BN, Orion, America). Part of the air-dried and primarily sieved samples were ground and then passed through a 0.25-mm sieve for further analysis of the C, N and P. Soil organic C (SOC) was determined by the Walkley–Black method of dichromate oxidation [30]. Soil total N was quantified by the semimicro Kjeldahl method [31]. Soil total P was measured by using the Alkali-molybdenum antimony colorimetric method [32]. To determine available nitrogen and phosphorus, we used the method consisting of diffusion of alkali solution and extraction of hydrochloric acid and sulfuric acid [31].

### Nutrient resorption efficiency and C:N:P stoichiometry

In this study, the nutrient resorption was expressed in terms of its efficiency. We calculated nutrient resorption efficiency by using the nutrient concentrations in the green (Gr) and senesced (Sen) leaves based on leaf mass [1], and corrected the calculation by using mass loss correction factors MCLF) of 0.741 for coniferous forest [8]. We use the following formula:

$$RE=(1-\frac{X_{Sen}}{X_{Gr}}MLCF)\times 100$$

X_Gr_, nutrient concentration in green leaves;
X_Sen_, nutrient concentration in senesced leaves.

Ecological stoichiometry of the differently-aged *L*. *kaempferi* plantations was calculated as mass ratios of C:N, C:P and N:P in the leaves and then compared with previously published results.

### Statistical analysis

We employed one-way analysis of variance (ANOVA) to test for differences in the leaf nutrient concentration, nutrient resorption efficiency, stoichiometry, and soil property among the four different aged plantations. The multiple comparisons of means were determined via the least significant difference (LSD) test at α = 0.05. All the data were analyzed in Excel 2010 and SPSS 16.0. If the age effect was significant, post-hoc multiple comparisons were subsequently made using Turkey HSD test. The resorption rates of N and P, and the ratio of C:N:P in the green and senesced leaves were analyzed in bivariate Pearson correlations.

### Ethics statement

Both leaf and soil samples used in the experiment have been collected in the Qinling Mountains, managed by Xiaolongshan Forestry Experiment Bureau of Gansu Province, China. The Experiment Bureau responsible for this protected area gave permission to conduct the study on this site. No specific permissions were required as the study did not involve endangered or protected species.

## Results

### Soil properties

Except for soil pH, there were significant differences (*p* < 0.05) in the SOC, total N, total P, available P and available N at the soil depth of 0–20 cm among the different aged plantations (Table 2). Among all soils of different aged plantations, the soil of 15-year-old plantation had the greatest values in these chemical components. The results indicated that the soil chemical properties and nutrient availability did not show a constant increasing or decreasing trend over plantation ages.

### Nutrient concentrations of the green and senesced leaves

**Fig 1:**
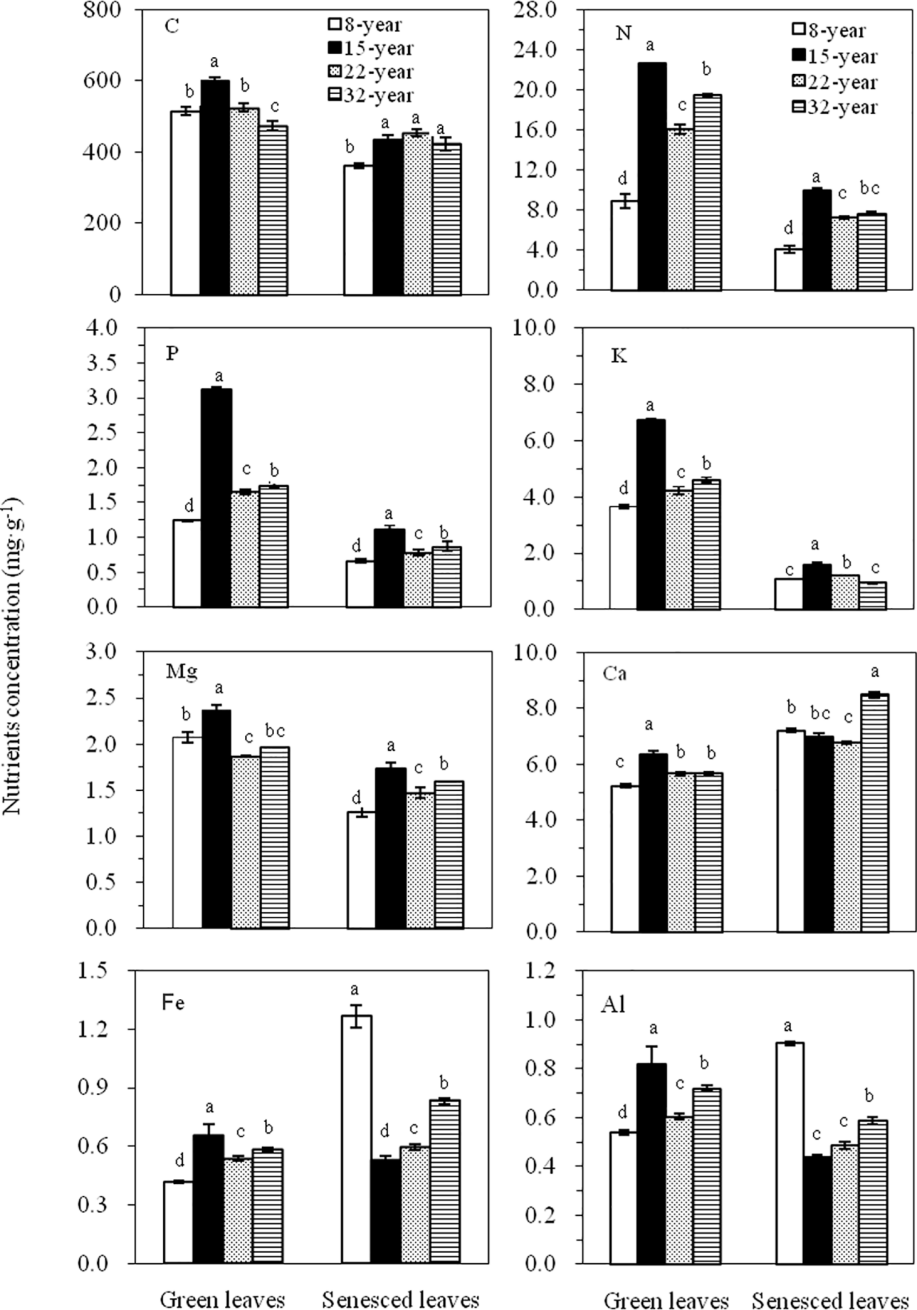
Nutrient concentrations in both green and senesced leaves in four aged plantations of *L*. *kaempferi*. Error bars represent the standard deviations of the means (n = 6). Different letters denote significant differences in nutrient concentrations among the four different-aged plantations of *L*. *kaempferi* (one-way ANOVAs, n = 6).

The carbon (C) concentrations in the green leaves ranged between 524.5 mg**·**g^-1^ in the 32-yr-old plantation and 600.4 mg**·**g^-1^ in the 15-yr-old plantation, showing a ranking order among the different aged plantations as 15-yr > 8-yr > 22-yr > 32-yr. In the senesced leaves, the C concentrations ranged from 360.68 to 413.27 mg**·**g^-1^, which were significantly lower than that in green leaves of the corresponding aged plantations. Meanwhile, the N and P in the green leaves of different aged plantations were in a concentration range of 8.83–22.63 mg**·**g^-1^, and 1.24–3.13 mg**·**g^-1^, respectively, which values were significantly higher than those in the senesced leaves of four different plantations. The concentrations of both N and P in either the green leaves or the senesced leaves showed a same rank-order across the plantation ages: 15-yr > 32-yr > 22-yr > 8-yr-old (Fig 1).

For the metal elements, the concentrations of K, Ca, Fe, and Al in the green leaves had the same rank-order among different aged plantations: 15-yr > 32-yr > 22-yr > 8-yr-old (Fig 1), whereas the concentration of Mg showed a slightly different rank-order: 15-yr > 8-yr > 32-yr > 22-yr-old (Fig 1). Compared between the green and the senesced leaves, the concentrations of K and Mg in the green leaves were significantly higher than those in the senesced leaves, but the Ca concentration in the green leaves was significantly lower than that in senesced leaves in all plantations. Fe concentration in the green leaves of four plantations was significantly lower than those in the senesced leaves except in the case of 15 yr-old plantation, while Al concentrations in the green leaves of four plantations was significantly higher than those in the senesced leaves except for 8 yr-old plantation. In addition, except for a limited number of nutrients, most of their concentrations in both the green and senesced leaves were significantly different among the studied plantation ages (Fig 1). The nutrient concentrations of N, P, K, Ca, Mg, Al, and Fe in both green and senesced leave did not show regular changes as the age changes of *L*. *kaempferi* plantations. Moreover, there were evidently positive relationships among the all studied elemental concentrations in the green leaves of *L*. *kaempferi*, with highly significant correlation coefficients found for them (*p*< 0.01) (Table 3).

**Table 3:** Simple linear Pearson correlations between the nutrient concentrations in the green (Gr) and senesced (Sen) leaves of *L*. *kaempferi*.

	Leaves	N	P	K	Ca	Mg	Fe	Al
**C**	Gr Sen	0.389 0.751**	0.763** 0.500	0.740** 0.333	0.731** —0.132	0.800** 0.634*	0.464 0.916**	0.486 0.901**
**N**	Gr Sen		0.821** 0.907**	0.842** 0.631*	0.899** 0.010	0.404 0.948**	0.993** 0.907**	0.941** —0.912**
**P**	Gr Sen			0.994** 0.725**	0.964** 0.032	0.814** 0.898**	0.994** —0.695*	0.909** —0.702*
**K**	Gr Sen				0.970** —0.638*	0.803** 0.499	0.881** —0.580*	0.924** —0.513
**Ca**	Gr Sen					0.690* 0.190	0.935** 0.239	0.916** 0.119
**Mg**	Gr Sen						0.476 —0.776**	0.651* —0.808**
**Fe**	Gr Sen							0.956** 0.976**

* *p* < 0.05.

** *p* < 0.01.

### Nutrient resorption efficiency

**Fig 2:**
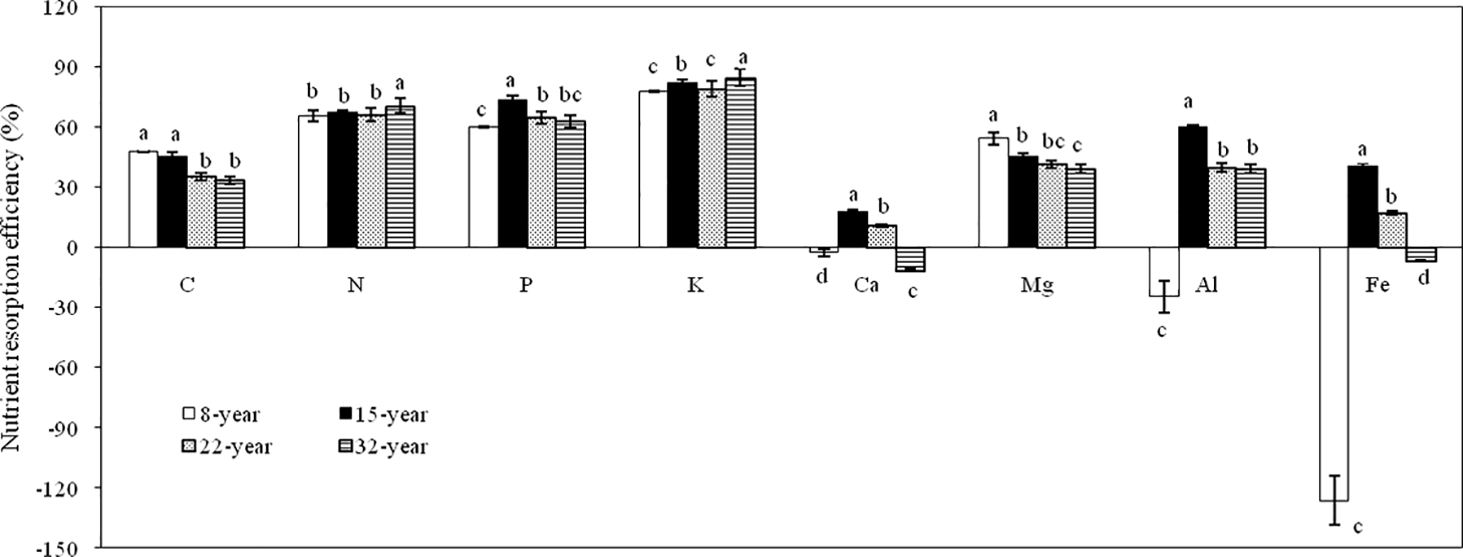
Nutrient resorption efficiency of leaves for four aged plantations of *L*. *kaempferi*. Different letters denote significant differences in nutrient resorption efficiency among the four different-aged plantations of *L*. *kaempferi* (one-way ANOVAs, n = 6).

The N-resorption efficiency (NRE) ranged from 65.61% to 70.69% in four different aged plantations of *L*. *kaempferi* (Fig 2). The highest NRE occurred in the 32-yr-old plantations, while roughly equivalent NRE was observed in the other three plantations (*p* < 0.05) (Fig 2). The P resorption efficiency (PRE) ranged between 60.34% and 73.47% for four different aged plantations. The 15-yr-old plantation had a PRE value of 73.74%, which was much higher than that in 8-, 22-, and 32-yr-old plantations with corresponding PRE value of 60.34%, 64.70% and 62.82%. (*p* < 0.05) (Fig 2). For the K and Mg in leaves from four plantations, the positive RE values ranged from 77.65% to 84.72% and from 39.39% to 54.34%, respectively, (Fig 2). The mean positive RE values of N, P, K, and Mg for four plantations showed the rank order of K (80.89%) > N (67.42%) > P (65.34%) > Mg (41.16%), which revealed the resorption activities of the four nutrients. However, positive REs of Ca and Fe were only found in leaves of both 15- and 22-yr old plantations (Fig 2). This demonstrated that N, P, K, and Mg had a higher mobility than Ca and Fe for all examined trees in four of the plantations (Fig 2). Meanwhile, a positive RE of Al was found in the 15-, 22-, and 32-yr-old plantations, but not in the 8-yr-old plantation (Fig 2).

In particular, most of the nutrient REs in present study did not change regularly along the plantation ages (Fig 2). Furthermore, the nutrient REs of the eight elements examined were positively related to their corresponding concentrations in the green leaves, though negatively related to their concentration in the senesced leaves apart from N and P (Table 4). NRE was negatively correlated with PRE as it was shown by Spearman's correlation coefficiency (r = —0.003, p > 0.05) in *L*. *kaempferi* (Table 5).

**Table 4:** Correlations between the nutrient resorption efficiency and the nutrient concentrations in the green and senesced leaves of *L*. *kaempferi*.

Leaves	C	N	P	K	Ca	Mg	Fe	Al
Gr	0.602*	0.515	0.963**	0.527	0.681*	0.361	0.915**	0.825*
Sen	—0.588*	0.332	0.860**	—0.052	—0.833**	—0.644*	—0.985**	—0.980**

* *p* < 0.05.

** *p* < 0.01.

**Table 5:** Correlations between the NRE or PRE and the C:N:P stoichiometric characteristics in green and senesced leaves.

	PRE	C:N_Gr_	N:P_Gr_	C:P_Gr_	C:N_Sen_	N:P_Sen_	C:P_Sen_
**NRE**	—0.003	—0.671*	0.579*	—0.423	—0.412	0.265	—0.284
**PRE**		—0.552	0.649*	—0.862**	—0.790**	0.919**	—0.724**
**C:N**_**G**_**r**			—0.853**	0.842**	0.933**	—0.792**	0.426
**N:P**_**G**_**r**				—0.895**	—0.891**	0.850**	-0.692*
**C:P**_**G**_**r**					0.963**	—0.980**	0.804**
**C:Nsen**						—0.953**	0.639*
**N:Psen**							—0.772**

* *p* < 0.05.

** *p* < 0.01.

### Stoichiometric ratios

**Fig 3:**
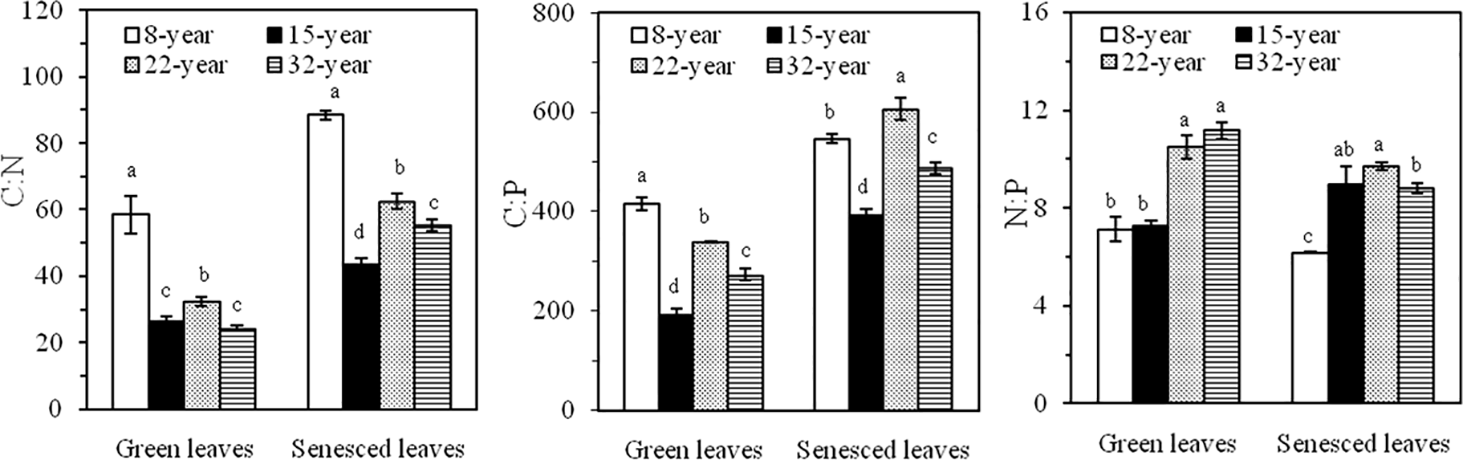
C:N:P stoichiometric characteristics in the green and senesced leaves for four aged plantations of *L*. *kaempferi*. Lowercase letters in the same column indicate a significant difference at α = 0.05. Error bars represent the standard deviations of the means (n = 6).

The results showed that the C:N and C:P ratios in both green and senesced leaves were significantly different (*p* < 0.05) for all the studied plantations (Fig 3). Conversely, there were no significant differences between the 8- and 15-yr-old plantations nor between the 22- and 32-yr-old plantations (*p* < 0.05) for the N:P ratio (Fig 3). The sampled trees of *L*. *kaempferi* had a C:N ratio which ranged from 25.52 to 58.54 in the green leaves, and from 43.64 to 88.41 in the senesced leaves (Fig 3). The C:P ratio ranged from 192.17 to 414.98 in the green leaves, and from 391.15 to 605.91 in the senesced leaves (Fig 3). The N:P ratio ranged from 7.12 to 11.18 in green leaves, and from 6.18 to 9.19 in the senesced leaves (Fig 3). Generally, both ratios of C:N and C:P in the green and senesced leaves from all studied trees were ranked 8-yr > 22-yr > 32-yr ≥ 15-yr for plantation age, and both ratios in the green leaves were lower than those in the senesced leaves for each plantation (Fig 3).

The relationship between the stoichiometric ratio and the nutrient RE were analyzed in the present study, for both green and senesced leaves. A significantly negative relationship between NRE and C:N ratio was only found in the green leaves (*r* = —0.671, *p* < 0.05), while negative correlation between the PRE and C:P ratio was significant for both the green (*r* = —0.862, *p* < 0.01) and senesced leaves (*r* = —0.724, *p* < 0.01). Among the stoichiometric ratios, C:N and C:P were positively and significantly correlated in both the green (*r* = 0.842, *p* < 0.01) and senesced leaves (*r* = 0.639, *p* < 0.05), whereas N:P was significantly negatively correlated with either C:N or C:P in both the green and senesced leaves (Table 5).

## Discussion

### Nutrient concentrations in green and senesced leaves

In plants, nutrient concentrations in leaves are often influenced by many biological and abiotic factors [28]. In this study, we addressed the question whether the nutrient utility and conservation of *L*. *kaempferi* might be affected by plantation age. We found that the nutrient concentrations of C, N, P, K, Ca, Mg, Fe, and Al in the green leaves of *L*. *kaempferi* did not change along their plantation ages, which indicated that the plantation age was not a key factor to influence nutrient concentrations in leaves under the same geographical condition. Moreover, the nutrient concentrations of all measured showed a same rank order of 15-yr > 32-yr > 22-yr > 8-yr in the green leaves except for Mg, indicating a certain proportion or balanced relationship of elements occurred in each plantation. This finding was further authenticated by the positive correlations among the element concentrations in the green leaves (Table 2). The concentrations of all elements in the green leaves peaked in the 15-yr-old plantation, which probably due to higher soil chemical components of the 15-yr-old plantation as compared with that of the other plantations (Table 2).

In the present study, the mean C concentration (503.01 mg·g^-1^) for four *L*. *kaempferi* plantatioins is much higher than 464 mg·g^-1^, mean value of C concentration in the leaves of terrestrial plants worldwide, but is equivalent to that of *L*. *principis-rupprechtii* growing in the region of the Qinling Mountains (472.5–527.41 mg·g^–1^) [24, 33]. This finding indicates that the fast-growing larch has a similar carbon storage capacity in their green leaves under the same climatic condition. In addition, conifers have many kinds of C-rich secondary compounds, such as lignin, tannins and waxes, which could contribute to higher C storage in their leaves [34]. On the other hand, the green leaves had the higher C concentration than the senesced ones, which may be caused by leaching of soluble carbon in leaves by heavy rainfall.

According to Marschner [35] and Han *et al* [36], the physiological concentrations of N, P, K, Ca, Mg, and Fe needed for adequate plant growth are 15, 2, 10, 5, 2, and 0.1 mg·g^-1^, respectively. In the present study, these threshold values were used to evaluate the relative requirement limitation of multiple nutrients to *L*. *kaempferi* growth. We noticed that only in the 15-yr old plantation did the nutrient concentrations of N, P, Ca, Mg, and Fe meet the above physiological requirements (Fig 1). However, in the 8-, 22-, 32-yr old plantations, the mean concentrations of P (1.54 mg·g^-1^) and K (4.17 mg·g^-1^) did not meet this physiological requirements, while N (14.74 mg·g^-1^), Mg (1.97 mg·g^-1^) and Ca (5.52 mg·g^-1^) were close to the requirement, but Fe (0.51 mg·g^-1^) was much higher (Fig 1). For all the aged plantations, the mean concentrations of N (16.7 mg·g^-1^), K (4.81 mg·g^-1^), Ca (5.73 mg·g^-1^), and Al (0.67 mg·g^-1^) in the green leaves were obviously lower than those reported averages of Chinese or global plants [36–39], whereas P concentration (1.94 mg·g^-1^) were higher than the Chinese average (1.41 mg·g^-1^), but close to the global one (1.99 mg·g^-1^) [33, 36, 38]. The mean concentration of Fe (0.55 mg·g^-1^) exceeded the corresponding Chinese or global averages, whereas Mg concentration (2.07 mg·g^-1^) was closer to [36, 37]. These results suggested that a relative limitation of multiple nutrients exists for *L*. *kaempferi* in the Qinling Mountains.

In senesced leaves, the N, P, K, and Mg concentrations were lower than those in green leaves, but vice-versa for Ca (Fig 1). This result could be explained by the relatively higher mobility of N, P, K, and Mg than Ca that functions as a structural element in plants [40, 41]. For the elements of Fe and Al, unexpectedly the highest concentration in senesced leaves was concurrently found in the 8-yr old plantation, which increased by 3.5-fold and 1.7-fold, respectively, compared to their corresponding concentration in green leaves (Fig 1). The most likely reason for highest Fe level in the 8-yr-old plantation is that Fe concentration in leaves of *L*. *kaempferi* growing in the Qinling region, ranged from 0.42 to 0.66 mg g-1, was much higher than its physiological requirement concentration (0.1 mg·g^-1^) to plant growth. This suggests that the accumulation of Fe element happened in *L*. *kaempferi*. In addition, due to having small biomass for the young aged plantations (i.e. 8-yr-old *L*. *kaempferi*), mass accumulated trace element Fe in leaves was not transferred to other organs of plants before leaves senesced, thus making most of Fe still preserved in senesced leaves. But with the leaves withered, the accumulated Fe was then removed from plant body, thereby realized the balance adjustment between Fe and other elements in plants. On the other hand, correlation analysis revealed that the concentrations of Al and Fe in green and senesced leaves of *Larix kaempferi* are positively correlated, with correlation coefficients being as high as 0.956 and 0.976 (Table 5), respectively, indicating that there is a coordinating role between these two elements in *L*. *kaempferi* in the Qinling Mountains. This might be the main reason for high Al content in the senesced leaves of *L*. *kaempferi*.

### Nutrient resorption efficiency

In present study, nutrient REs of seven elements did not show a regular change trends along the *L*. *kaempferi* plantation ages, which was inconsistent with what has reported in other plants [7, 42, 43]. This is largely due to the nutrient concentrations of seven elements in both green and senesced leaves of *L*. *kaempferi*, which showed unregular change trends along the plantation ages in the Qinling Mountains. We also found that the average REs for N (67.42%), P (65.34%), K (80.89%) and Mg (41.16%) were higher than the respective global values [1, 8, 44]. On the one hand, this is because the uncorrection of RE by using the mass loss correction factor (MLCF) led to low real RE value in previous studies [8, 41]; on the other hand, this is also because a relatively more nutrient resorption in *L*. *kaempferi* under the limitation of multiple nutrients in the Qinling Mountain, in agreement with a leaf-economics perspective.

In this study, we found that positive REs happened in leaves of both 15- and 22-yr-old plantations for Fe, and in 15-, 22- and 32-yr-old plantations for Al, whereas a negative RE in 8-, 32-yr-old plantations for Fe and in 8-yr-old plantations for Al, respectively. The former is accord to the reports that there were significantly different RE values for Fe in deciduous trees [45]. The latter implies that the detoxification of Al in *L*. *kaempferi* has not yet occurred despite the acidic soil of the plantations at our site (Table 2). Furthermore, such lacking a single pattern for Fe and Al resorptions hints their complex process operation in the *L*. *kaempferi* plantation dynamics.

Although some studies reported a negative relationship between the concentration and RE of nutrients in green leaves, suggesting that plants in low-input systems are more nutritionally efficient [8, 12, 46]. Meanwhile, many studies did not discern any clear relationships as such [1, 5, 44, 47]. Our results for *L*. *kaempferi* in this study showed that a positive relationship between the RE and concentration of nutrients exists in green leaves, which is agreed with findings by Pastor-Pastor *et al* [48].

### Stoichiometric ratios

At the current study, mean C:N and C:P ratios (35.44 and 304.49, respectively) in the green leaves greatly exceed the corresponding global averages (22.5 and 232, respectively) [32], yet they are much lower than those found in the senesced leaves (62.49 and 507.77, respectively) of *L*. *kaempferi*. On the one hand, the C concentration in the green leaves of *L*. *kaempferi* is much higher than that of terrestrial plants worldwide, which contributes to elevated C:N and C:P ratios in *L*. *kaempferi*. On the other hand, the high stoichiometric ratios in *L*. *kaempferi* are partly due to the relatively low concentrations of N and P in its green leaves. In addition, both the C:N and C:P ratios in green and senesced leaves followed the same rank order of plantation age (8-yr > 22-yr > 32-yr > 15-yr) that was the inverse followed by the N and P concentration in green leaves (8-yr < 22-yr < 32-yr < 15-yr). This finding confirms that the concentrations of N and P in the green leaves are the major determinants for the C:N and C:P ratios in *L*. *kaempferi*.

In addition, the N:P ratio in leaves is considered important for the structuring and functioning of plant communities; hence, it is frequently used as an indicator to assess nutrient availability to plants [49–51]. In this study, mean N:P ratio for sampled trees was 9.00 and 8.42 in their green and senesced leaves, respectively; this values are much lower than those of China’s flora and global plants[6, 38, 52]. The lower mean N:P ratio in the senesced leaves indicates that the plant could resorb more N than P during senescence. Furthermore, Koerselman and Meuleman (1996) found that if the N:P ratio was <14, plant growth was limited by N; if the N:P ratio was 14–16, plant growth was limited by both N and P; conversely, if the N:P ratio >16, then plant growth was limited by P [51]. So when applied to the current study, the results—an N:P ratio mostly of 9.00 vs. the 14 threshold above—suggest that N is a limiting factor for *L*. *kaempferi* growth in the Qinling Mountains area.

Given that litter on the forest floor acts as an input-output system of nutrients, the rates at which litter falls and decays will contribute to the regulation of nutrient cycling, as well as to soil fertility and primary productivity in forest ecosystems [3, 53–56]. Foliar litter that has lower C:N and lignin:N ratios is more susceptible to decomposition, because the microbial growth and invertebrate digestion are better stimulated under such conditions [49]. In contrast, the leaves of *L*. *kaempferi*, with their high C:N ratio and low N concentration, should indirectly hinder leaf litter decomposition. If so, nutrient turnover and cycling, as well as the soil fertility and primary productivity for the forest ecosystem, might be negatively influenced by *L*. *kaempferi* leaf litter in the Qinling Mountains.

## Conclusions

The present study suggests the nutrient concentrations of C, N, P, K, Ca, Mg, Fe, and Al in the green and senesced leaves do not change with the plantation age of *L*. *kaempferi* in the Qinling Mountains. Most nutrients, except for Fe, in these *L*. *kaempferi* are either relatively lower than or close to the physiological concentrations required, thus suggesting a relative limitation of multiple nutrients for *L*. *kaempferi* growth. Much higher concentrations of nutrients in the leaves of the 15-yr old plantation could be attributed to the relatively higher content of available nutrients in that plantation’s soil. The low N:P ratio for this species further suggests that the plantation growth of *L*. *kaempferi* was mainly limited by N nutrient. Thus, nitrogen fertilizer should be added in a timely way to meet the nutritional needs for *L*. *kaempferi*. The nutrient resorption efficiency (RE) of N, P, K and Mg revealed resorption activity, while mineral nutrients Ca and Fe tended to accumulate in senesced leaves. The absence of a single resorption pattern for Ca, Fe or Al, however, in four different aged *L*. *kaempferi* plantations indicates that these processes of nutrient resorption are quite complicated, and it is unlikely that one factor governs the nutrient resorption patterns alone. Overall, the nutrient REs of all the measured elements do not change with plantation age, though they were positively related to their respective concentrations in the green leaves of *L*. *kaempferi*. Based on the RE of Al, we conjecture that Al detoxification did not occur in *L*. *kaempferi* under the present study conditions, even though Al toxicity is common in certain acidic soils. Nevertheless, *L*. *kaempferi* leaves that have a higher C:N ratio and lower N concentration will ultimately bring about decrease of rates of leaf litter decomposition. This may have a negative influence on the nutrient turnover and cycling, as well as the soil fertility and primary productivity, of plantation forest ecosystems in the Qinling Mountains. Appropriate human interventions, such as applying nitrogen fertilizer to the present plantations or establishing mixted forest types of coniferous and broad-leaved forest, should be pursued to ensure a balance of ecological and economic benefits as provided by the *L*. *kaempferi* plantation forest ecosystem into the future.

## Supporting information

S1 Fig The geographical position of study area. (TIF)
