# Supplementary figures and images for: Nutrients resorption and stoichiometry characteristics of different-aged plantations of Larix kaempferi in the Qinling Mountains, central China
Source: PLoS One. 2017 Dec 15;12(12):e0189424. doi: 10.1371/journal.pone.0189424 (PMC5731737; doi:10.1371/journal.pone.0189424)

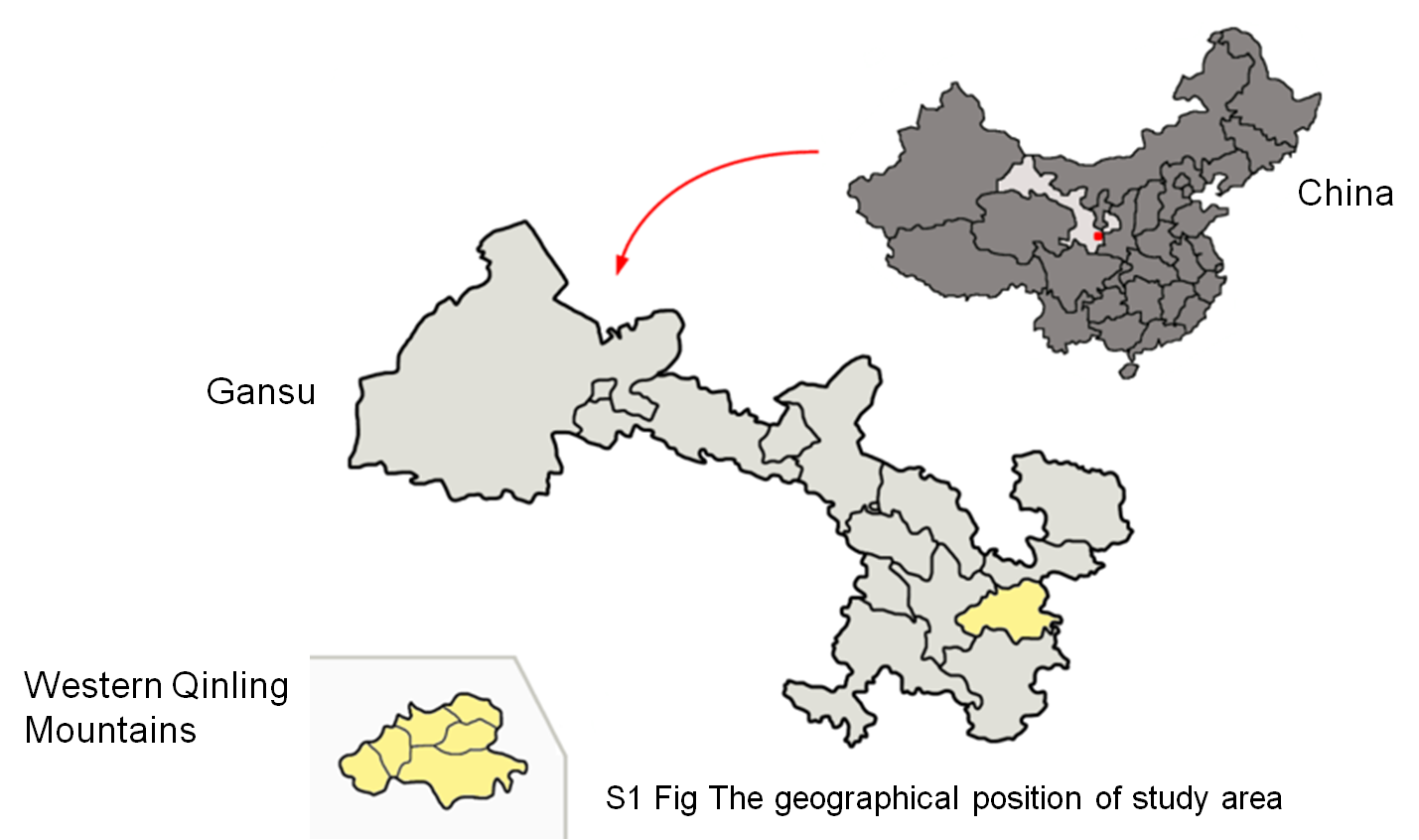

Supplement: S1 Fig — (TIF) [file pone.0189424.s001.tif]
